# CRISPR/Cas9-mediated simultaneous targeting of GmP34 and its homologs produces T-DNA-free soybean mutants with reduced allergenic potential

**DOI:** 10.3389/fpls.2025.1612747

**Published:** 2025-08-01

**Authors:** Dongwon Baek, Byung Jun Jin, Mi Suk Park, Ye Jin Cha, Tae Hee Han, Ye Na Jang, Su Bin Kim, Sang In Shim, Jong-Il Chung, Hyun Jin Chun, Min Chul Kim

**Affiliations:** ^1^ Plant Molecular Biology and Biotechnology Research Center, Gyeongsang National University, Jinju, Republic of Korea; ^2^ Division of Applied Life Science (BK21 Four), Gyeongsang National University, Jinju, Republic of Korea; ^3^ Institute of Agriculture and Life Science, Gyeongsang National University, Jinju, Republic of Korea

**Keywords:** soybean, allergen, CRISPR/Cas9, genome editing, GmP34 homologs

## Abstract

Soybean (*Glycine max* L.) P34 (GmP34) is a prominent allergenic seed protein belonging to the papain-like cysteine protease family. To mitigate its allergenic potential, we implemented a CRISPR/Cas9-based genome editing strategy targeting *GmP34* along with its two highly similar homologs, *GmP34h1* and *GmP34h2*, in the soybean cultivar Williams 82. Phylogenetic analysis and domain characterization identified GmP34h1 and GmP34h2 as the closest homologs to GmP34, with conserved allergenic peptide motifs. Gene expression profiling revealed similar expression patterns of all three genes during seed maturation, indicating potential functional redundancy. Two multiplex CRISPR/Cas9 constructs were designed to simultaneously target *GmP34*/*GmP34h1* and *GmP34*/*GmP34h1*/*GmP34h2* genes, respectively. Transgenic genome editing plants were generated via *Agrobacterium*-mediated transformation, and targeted mutagenesis was confirmed by genomic PCR and deep sequencing. Loss of GmP34 protein in edited lines was further validated through western blot analysis. Using this strategy, we successfully generated *GmP34* single, *GmP34*/*GmP34h1* double, and *GmP34*/*GmP34h1*/*GmP34h2* triple mutants. This study highlights the utility of multiplex genome editing in silencing soybean allergenic gene and its homologs. Ongoing analyses of allergenicity in these edited lines aim to provide a genetic foundation for the development of hypoallergenic soybean cultivars through precise genome engineering.

## 1 Introduction

Soybean (*Glycine max* [L.] Merr.) is a globally important crop, valued for its high-quality protein and oil, which are widely used in both human and animal diets. However, food products derived from soybeans can provoke allergic reactions in sensitive individuals due to specific seed storage proteins that function as allergens. Several of these seed proteins have been identified as allergenic, exhibiting immunoglobulin E (IgE) binding activity and containing IgE/IgG-binding epitopes (Riascos et al., 2009; Cabanillas et al., 2017; Kern et al., 2018; Wiederstein et al., 2023).

Soybean is recognized as one of the eight major food allergens (Cordle, 2004). To date, 16 soybean proteins with immunoglobulin E (IgE) binding activity have been identified as allergens involved in immune-mediated allergic responses (Wilson et al., 2008). According to the World Health Organization (WHO) and the International Union of Immunological Societies (IUIS), eight of these proteins—designated Gly m 1 to Gly m 8—are officially classified as soybean allergens (http://www.allergen.org/index.php). Soybean allergens are categorized into two classes—class 1 and class 2—based on differences in sensitization routes (Maruyama et al., 2018; Matsuo et al., 2020). Class 1 food allergens are primarily associated with direct sensitization through ingestion, particularly in early childhood, and can cause symptoms such as urticaria, diarrhea, vomiting, atopic dermatitis, and anaphylaxis (Matsuo et al., 2020; Wiederstein et al., 2023). This group includes Gly m 5 (7S globulin), Gly m 6 (11S globulin), Gly m 7 (seed biotinylated protein), Gly m 8 (2S albumin), Gly m KTI (Kunitz-type trypsin inhibitor), Gly m BBI (Bowman–Birk inhibitor), Gly m Bd 30K/GmP34 (thiol protease-like protein), and Gly m Bd 28K (vicilin-like protein) (Matsuo et al., 2020; Wiederstein et al., 2023). Class 2 food allergens are associated with secondary sensitization due to cross-reactivity with other legumes or pollen allergens, often leading to comorbid allergic responses (Matsuo et al., 2020; Wiederstein et al., 2023). This group includes Gly m 1 (hydrophobic seed protein), Gly m 2 (defensin), Gly m 3 (profilin), and Gly m 4 (a pathogenesis-related protein belonging to the PR-10 family, also known as starvation-associated message 22, SAM22). These allergens are commonly linked to oral allergy syndrome, airway constriction, breathing difficulties, and anaphylaxis accompanied by facial swelling (Matsuo et al., 2020; Wiederstein et al., 2023). Notably, Gly m 1 and Gly m 2 are found in the soybean hull and function as potent respiratory allergens (Pi et al., 2021).

Among the recognized soybean allergens, Gly m 4, Gly m 5, Gly m 6, Gly m Bd 28K, and Gly m Bd 30K are immunodominant proteins identified as major contributors to soybean allergenicity (Wiederstein et al., 2023). Gly m 4, a pathogenesis-related 10 (PR-10) protein, is prevalent in smoothly processed soy products such as soymilk and exhibits strong cross-reactivity with the birch pollen allergen Bet v 1. This cross-reactivity can occasionally lead to severe allergic reactions, including anaphylaxis in individuals with birch pollinosis (Kosma et al., 2011; Asero et al., 2021; Finkina et al., 2022). Gly m 5 and Gly m 6, the major seed storage proteins belonging to the cupin superfamily, constitute 60%–80% of the total protein content in soybean seeds (Wang et al., 2014). Gly m 5, a β-conglycinin protein with a molecular weight of 180 kDa, comprises α, α’, and β subunits (Singh et al., 2015). Gly m 6, a 360 kDa glycinin protein, is the most abundant protein in soybean seeds and forms a hexameric structure composed of Gly m 1 to Gly m 5 subunits (Maruyama et al., 2001). Both Gly m 5 and Gly m 6 are clinically significant allergens known to trigger severe immune responses, including anaphylaxis (Holzhauser et al., 2009; Lu et al., 2018). Gly m Bd 28K is a vicilin-like protein belonging to the cupin superfamily, with a molecular weight of 26 kDa, and is isolated from the 7S globulin fraction (Xiang et al., 2004). Gly m Bd 30K, also known as GmP34 in soybeans, is a cysteine protease classified within the papain family. GmP34 is initially produced as a pre-pro-precursor protein with a molecular weight of 46–47 kDa, which undergoes processing through the removal of a 122-amino acid N-terminal signal peptide. The mature form, a 34 kDa protein, is ultimately localized in the protein storage vacuoles of soybean seeds (Kalinski et al., 1992; Ogawa et al., 1993). Despite being a relatively low-abundance seed protein—comprising less than 1% of the total seed protein—GmP34 is classified as a major allergen, as over 65% of soy-sensitive individuals exhibit allergic responses exclusively to this protein (Ogawa et al., 1993; Helm et al., 2000).

Several strategies have been investigated to reduce or eliminate GmP34 in soybean, including transgene-induced gene silencing (Herman et al., 2003), natural variant screening (Joseph et al., 2006; Bilyeu et al., 2009), and, more recently, genome editing via the clustered regularly interspaced short palindromic repeats (CRISPR)/CRISPR-associated protein 9 (Cas9) system (Sugano et al., 2020; Adachi et al., 2021). The first biotechnology-based approach to eliminate GmP34 in transgenic soybean plants employed a cosuppression-mediated gene-silencing technique (Herman et al., 2003). As an alternative strategy, researchers screened the USDA national soybean germplasm collection and identified two soybean accessions, PI603570A and PI567476, with significantly reduced levels of GmP34 protein (Joseph et al., 2006). These accessions were later found to carry a four-nucleotide insertion at the GmP34 start codon, which disrupts efficient translation (Bilyeu et al., 2009; Koo et al., 2013). More recently, both GmP34 single mutants and GmP34/Gly m Bd 28K double mutants were developed using CRISPR/Cas9-mediated genome editing (Sugano et al., 2020; Adachi et al., 2021). Although these approaches have shown promise, most studies have focused exclusively on the *GmP34* gene, without addressing its closely related homologs that may also contribute to allergenicity due to their sequence and functional similarity.

Recent advances in genomic resources and bioinformatic tools have enabled the identification and functional characterization of gene families with potential allergenic properties. In this study, we discovered two previously uncharacterized GmP34 homologs, GmP34h1 and GmP34h2, which exhibit high sequence similarity to GmP34 and contain conserved allergenic peptide motifs. Expression profiling revealed that all three genes are co-expressed during seed maturation, suggesting possible functional redundancy and shared roles in seed development and allergenicity.

To generate hypoallergenic soybean mutants, we employed multiplex CRISPR/Cas9-mediated genome editing to simultaneously target GmP34 and its homologs. By designing guide RNAs to induce mutations in all three genes, we successfully obtained *GmP34* single, *GmP34*/*GmP34h1* double, and *GmP34*/*GmP34h1*/*GmP34h2* triple mutants. These mutants were validated using insertion/deletion (InDel) PCR and targeted deep sequencing, and the absence of GmP34 protein was further confirmed through western blot analysis. This study represents the first report of simultaneous mutagenesis of *GmP34* allergenic gene and its closest homologs highlighting the effectiveness of multiplex genome editing for crop improvement, particularly in polyploid or genome-duplicated species such as soybean. We intend to evaluate the allergenicity of these edited lines in the further study to establish a foundation for the development of hypoallergenic soybean cultivars through precise genome engineering.

## 2 Materials and methods

### 2.1 *In silico* analysis

Multiple amino acid sequence alignments were generated using Clustal Omega (https://www.ebi.ac.uk/jdispatcher/msa/clustalo). Protein domains were identified and analyzed with PROSITE (https://prosite.expasy.org/) and PredictProtein (https://predictprotein.org/).

### 2.2 Plant materials and growth conditions

The soybean cultivar Williams 82 (cv. W82) served as the wild-type control in all experiments. Seeds were germinated in a growth chamber under long-day photoperiod conditions (16 h light/8 h dark) at 25°C, then transferred to a greenhouse and maintained under natural environmental conditions.

### 2.3 mRNA expression analysis

Seed samples were harvested from pods at the reproductive stage R6. Total RNA was isolated using the TRIzol reagent (Thermo Fisher Scientific, Waltham, MA, USA) according to the manufacturer’s instructions. Contaminating genomic DNA was eliminated using DNase I (Thermo Fisher Scientific), and first-strand cDNA was synthesized from 1 μg of RNA using SuperScript III Reverse Transcriptase (Thermo Fisher Scientific). Quantitative real-time RT-PCR (qRT-PCR) was conducted with gene-specific primers (**Supplementary Table 2**) using the QuantiSpeed SYBR No-Rox Kit (PhileKorea, Seoul, Korea). *GmPBB2* (Glyma.14G014800) was used as the internal reference gene. Relative expression levels were automatically calculated from triplicate reactions using the CFX Real-Time PCR Detection System and CFX Manager Software v2.0 (Bio-Rad, Hercules, CA, USA). All experiments were performed in a minimum of three independent biological replicates with three technical replicates per biological replicate. Statistical significance was determined using the Student’s *t*-test.

### 2.4 Western blot analysis

Total protein was extracted from soybean cotyledons using an extraction buffer containing 100 mM Tris-HCl (pH 7.5), 1 mM EDTA, 150 mM NaCl, 3 mM DTT, and 1 mM PMSF. One microgram of total protein per sample was resolved on a 15% SDS-PAGE gel and transferred onto Immobilon-P PVDF membranes (Merck Millipore, Co. Wicklow, Ireland). The membranes were incubated with a polyclonal anti-GmP34 antibody and visualized using an HRP-conjugated anti-rabbit IgG secondary antibody (Proteintech, Rosemont, IL, USA) in combination with ECL detection reagent (TransLab, Daejeon, Korea). As a loading control, five micrograms of protein were stained with Coomassie Brilliant Blue.

### 2.5 Guide RNA design and genome editing vector construction

Genomic sequences of *GmP34* (Glyma.08g116300), *GmP34h1* (Glyma.08g116400), and *GmP34h2* (Glyma.05g158600) were retrieved from the Phytozome v13 database (https://phytozome-next.jgi.doe.gov/). Candidate guide RNAs (gRNAs) were designed using CRISPR-P v2 (http://crispr.hzau.edu.cn/CRISPR2/) and CRISPR RGEN tools (http://www.rgenome.net/). Specifically, guide RNAs (gRNAs) with a GC content between 30% and 70% were initially selected using CRISPR-P v2. These candidates were further refined by identifying gRNAs with zero to three potential off-target mismatches across the genome, as predicted by Cas-OFFinder from RGEN Tools. Finally, we selected gRNAs that did not target the exon regions of any genes, except for our intended target genes. Three gRNAs—gRNA1, gRNA2, and gRNA3 were selected to simultaneously target either *GmP34* and *GmP34h1* (gRNA1/gRNA2) or all three genes (*GmP34*, *GmP34h1*, and *GmP34h2*) using the gRNA1/gRNA3 combination, with minimal predicted off-target activity (**Supplementary Table 1**). The constructs were assembled into the pECO201 binary vector using the Golden Gate cloning strategy (Oh et al., 2020). This vector features the *Arabidopsis ubiquitin6 (AtU6)* promoter for multi-tRNA-gRNA expression, the *NOS* promoter for *Bar* gene selection, and the CaMV *35S* promoter for expressing *Arabidopsis* codon-optimized *Cas9* (*acoCas9*). The gRNA sequences are listed in **Supplementary Table 1**.

### 2.6 *Agrobacterium*-mediated soybean transformation

Soybean W82 seeds were transformed using an *Agrobacterium*-mediated half-seed method, with minor modifications based on a previously published protocol (Kim et al., 2017). Seeds were surface-sterilized inside a desiccator for 5 min using chlorine gas generated from 1% sodium hypochlorite. Sterilized seeds were then germinated on germination medium in the dark for 20 h. Under sterile conditions, germinated seeds were halved longitudinally, and the seed coats were carefully removed. The half-seeds were then immersed in a suspension of *Agrobacterium tumefaciens* strain EHA105 harboring the CRISPR/Cas9 construct for 30 min at room temperature, followed by co-cultivation at 23°C for 5 h under a 16 h light/8 h dark photoperiod. After co-cultivation, explants were sequentially transferred to shoot induction medium and subsequently to root induction medium. Regeneration was carried out at 23°C under 16 h light/8 h dark conditions until the shoots exceeded 4 cm in height and roots reached lengths >5 mm.

### 2.7 Genome editing analysis and targeted deep sequencing

Genomic DNA was isolated from leaves of transformed soybean plants using the Exgene™ Plant SV Kit (GeneAll Biotechnology, Seoul, Korea). Transgenic lines were initially screened by PCR amplification of the *Bar* and *Cas9* genes using gene-specific primers (**Supplementary Table 2**). The PCR conditions were as follows: an initial denaturation at 95 °C for 10 min; 30 cycles of 95°C for 30 s, 58°C for 10 s, and 72°C for 30 s; followed by a final extension at 72°C for 10 min. Genomic regions targeted for editing were PCR-amplified for subsequent InDel detection and deep sequencing analysis. The PCR conditions for InDel detection were: 94°C for 5 min; 35 cycles of 94°C for 45 s, 60°C for 30 s, and 72°C for 1 min; followed by a final extension at 72°C for 10 min. Amplicons were further processed by incorporating adapter sequences via an additional round of PCR (Oh et al., 2020). The adapter PCR conditions were: 94°C for 3 min; 5 cycles of 94°C for 30 s, 50°C for 30 s, and 72°C for 1 min; followed by 25 cycles of 94°C for 30 s, 65°C for 30 s, and 72 °C for 1 min; with a final extension at 72°C for 10 min. Deep sequencing was carried out using the Illumina MiSeq platform (v2, 300-cycle; San Diego, CA, USA), and the resulting data were analyzed using Cas-Analyzer, part of the CRISPR RGEN Tools suite (http://www.rgenome.net/).

## 3 Results

### 3.1 Identification of *GmP34* homologs in soybean

**Figure 1:**
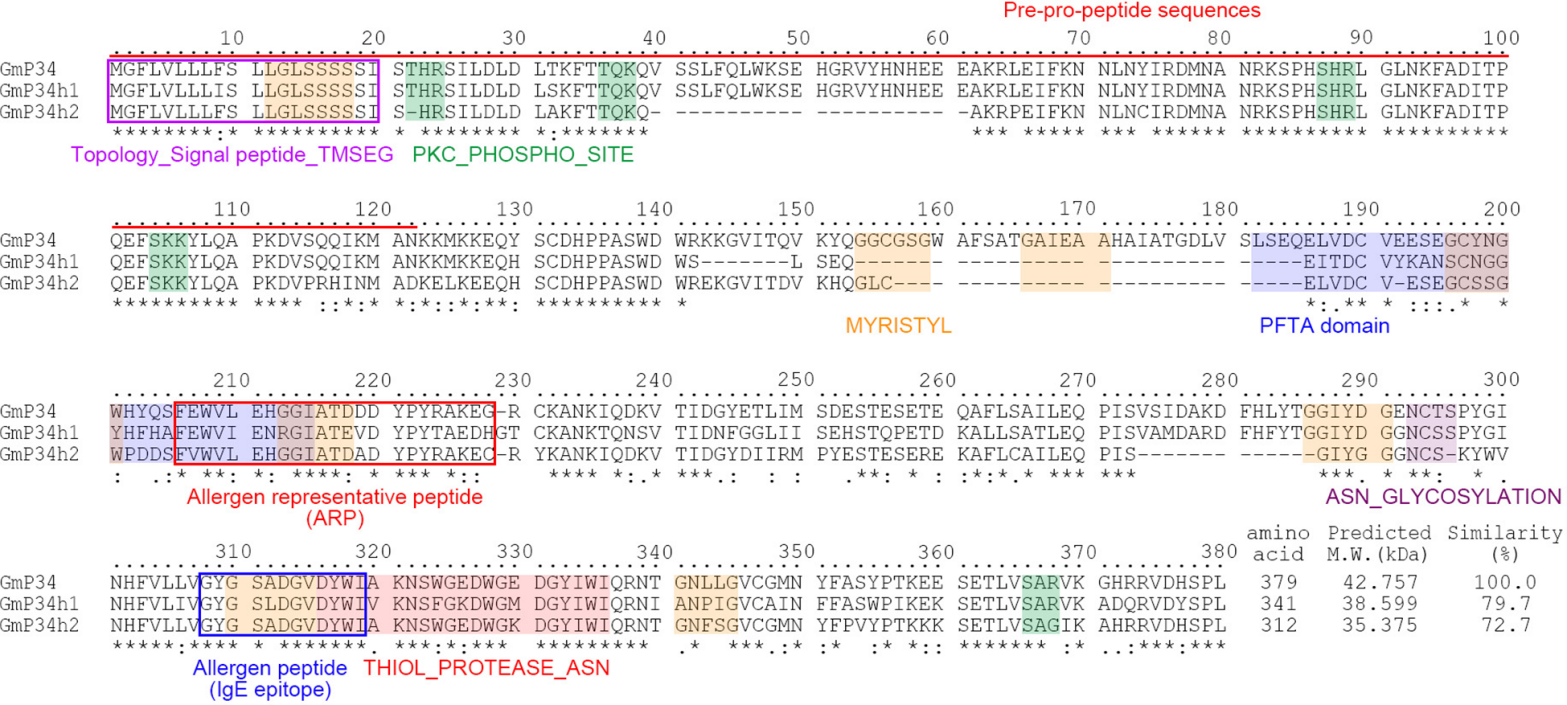
Amino acid sequence alignment and domain prediction of GmP34 and its homologs. Multiple sequence alignment of GmP34 (*Glyma.08G116300*), GmP34h1 (*Glyma.08G116400*), and GmP34h2 (*Glyma.05G158600*) was conducted using Clustal Omega. Asterisks indicate identical amino acid residues; dots denote conserved physicochemical properties. Functional domains were predicted using PROSITE (https://prosite.expasy.org/) and PredictProtein (https://predictprotein.org/). Color-coded boxes represent the following features: purple, signal peptide (TMSEG); red, allergen representative peptide (ARP); blue, IgE-binding epitope; orange, N-myristoylation site (MYRISTYL); green, protein kinase C phosphorylation site (PKC_PHOSPHO_SITE); navy, PFTA domain; purple (alternate), N-glycosylation site (ASN_GLYCOSYLATION); pink, thiol protease Asn active site (THIOL_PROTEASE_ASN). The red underline indicates pre-pro-peptide sequences.

The soybean *P34* gene (*GmP34*, Glyma.08G116300) encodes a papain-like cysteine protease and is recognized as one of the major seed protein allergens (Kalinski et al., 1990, 1992). Analysis of the soybean genome via the Phytozome database (https://phytozome-next.jgi.doe.gov/ identified approximately 100 proteins exhibiting sequence similarity to GmP34. Of these, the ten most closely related proteins were subjected to phylogenetic analysis, which identified two highly similar homologs: GmP34h1 (Glyma.08G116400) and GmP34h2 (Glyma.05G158600), sharing 79.7% and 72.7% amino acid similarity with GmP34, respectively (**Supplementary Figure 1**). GmP34 is initially produced as a pre-pro-protein and undergoes processing that includes the removal of a 122-amino acid N-terminal signal peptide, ultimately yielding the mature 34 kDa protein localized in the protein storage vacuoles of soybean seeds (Kalinski et al., 1992). Multiple amino acid sequence alignment and domain prediction using PROSITE (https://prosite.expasy.org/) and PredictProtein (https://predictprotein.org/) revealed that GmP34, GmP34h1, and GmP34h2 possess conserved structural motifs. These include endoplasmic reticulum signal peptides, protein kinase C phosphorylation sites, myristoylation sites, a PFTA domain, N-glycosylation sites, and a thiol protease Asn active site (**Figure 1**). Notably, allergen-associated motifs such as allergen representative peptides (ARPs) and IgE-binding epitopes were also conserved across all three homologs, suggesting a potential shared allergenic function (**Figure 1**).

### 3.2 Expression patterns of GmP34 homologs during soybean seed maturation

**Figure 2:**
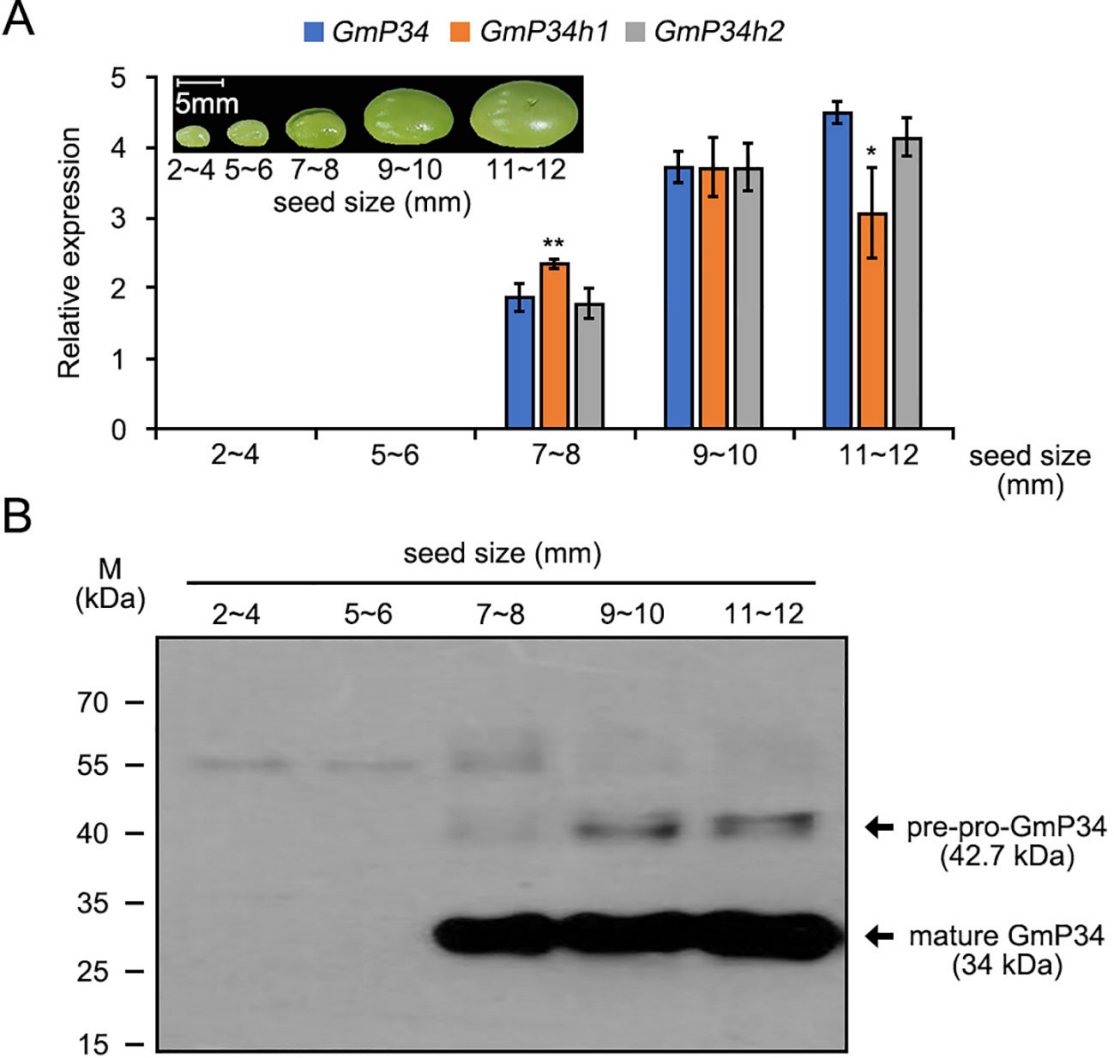
Expression dynamics of GmP34 and its homologs during soybean seed maturation. **(A)** Quantitative RT-PCR analysis of *GmP34*, *GmP34h1*, and *GmP34h2* transcript levels in developing seeds of W82 soybean at different developmental stages (2–4 mm, 5–6 mm, 7–8 mm, 9–10 mm, and 11–12 mm in length). *GmPBB2* was used as a reference gene. Error bars indicate standard deviation (SD) from three biological replicates. Asterisks denote statistically significant differences in expression relative to *GmP34* (*0.01 < *p* ≤ 0.05; **p ≤ 0.01, Student’s t-test). **(B)** Western blot analysis of GmP34 protein accumulation across seed maturation stages, detected using a polyclonal anti-GmP34 antibody.

Previous studies have shown that GmP34 is specifically expressed during soybean seed maturation (Kalinski et al., 1992; Koo et al., 2011, 2013). To investigate the expression profiles of the three *GmP34* homologs, we conducted qRT-PCR using gene-specific primers (**Supplementary Table 1**) on total RNA extracted from developing soybean seeds of various sizes (2–4 mm, 5–6 mm, 7–8 mm, 9–10 mm, and 11–12 mm). All three homologs exhibited comparable expression patterns, with transcripts first detectable in 7–8 mm seeds and progressively increasing with seed maturation. Notably, *GmP34h1* transcript levels declined more rapidly in 11–12 mm seeds compared to *GmP34* and *GmP34h2* (**Figure 2A**). These transcriptional patterns were further supported by western blot analysis using an anti-GmP34 polyclonal antibody (**Figure 2B**), suggesting functional similarity among the three proteins.

### 3.3 Development of soybean mutants targeting *GmP34* homologs using CRISPR/Cas9-mediated genome editing technology

**Figure 3:**
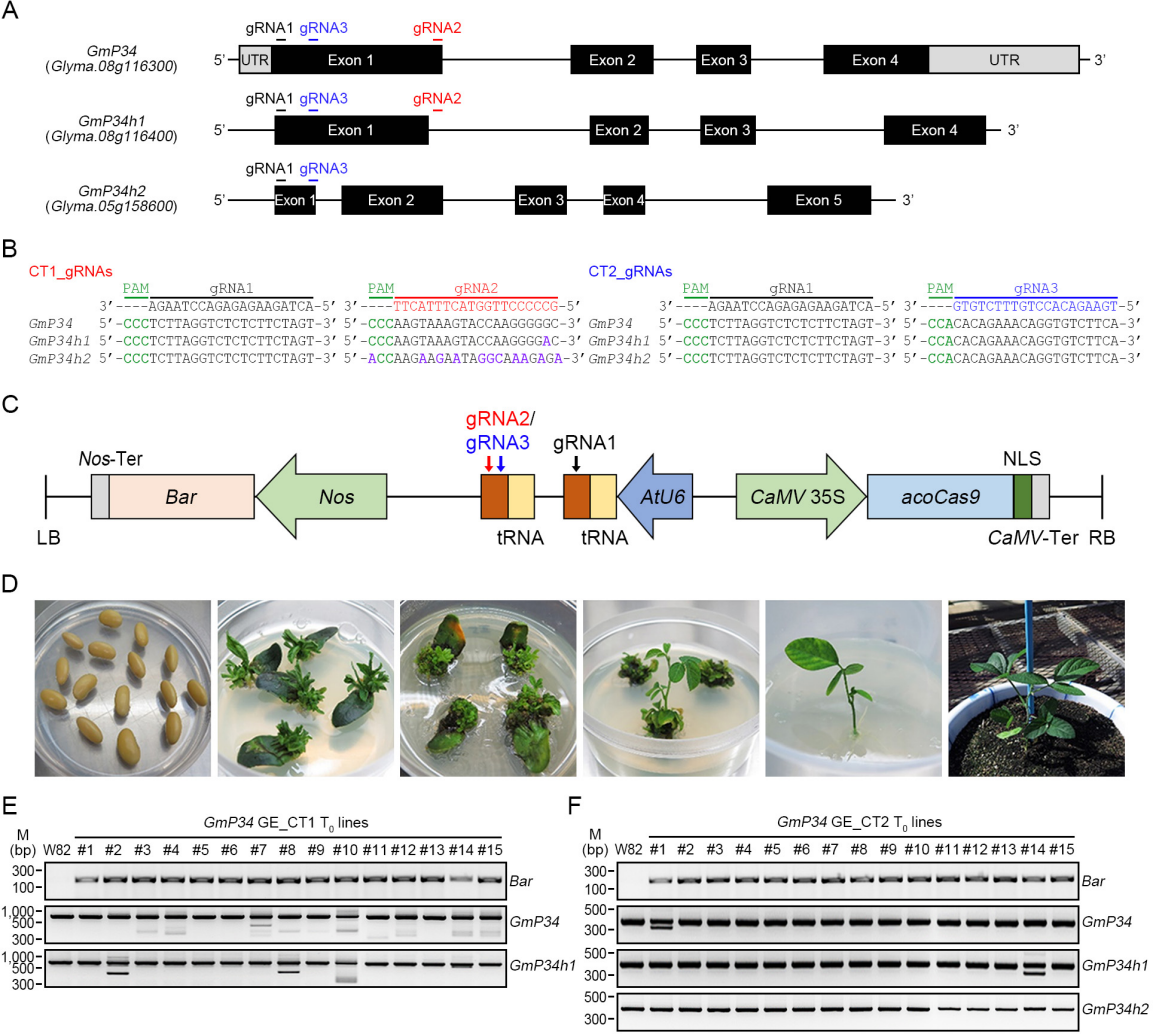
Generation of *GmP34* genome-edited (GE) soybean plants. **(A)** Schematic illustration of gRNA target sites within the *GmP34*, *GmP34h1*, and *GmP34h2* gene sequences. **(B)** Sequence alignment of gRNAs with their corresponding target regions in *GmP34*, *GmP34h1*, and *GmP34h2*. PAM sequences are highlighted in green. The target regions for gRNA1, gRNA2, and gRNA3 are shown in black, red, and blue, respectively. Mismatched nucleotides in *GmP34h1* and *GmP34h2* relative to the *GmP34* sequence are indicated in purple. **(C)** Schematic map of the binary vector used for co-expression of *Cas9* and gRNAs. The *Arabidopsis* codon-optimized *Cas9* (*acoCAS9*) was driven by the CaMV *35S* promoter, while gRNAs were expressed under the control of the *Arabidopsis U6* (*AtU6*) promoter. The gRNA1/gRNA2 combination was used to target *GmP34* and *GmP34h1*, while the gRNA1/gRNA3 combination was designed to target all three genes: *GmP34*, *GmP34h1*, and *GmP34h2*. NLS, nuclear localization signal; LB/RB, left and right T-DNA borders. **(D)** Workflow of *Agrobacterium*-mediated transformation in soybean, showing key stages from left to right: co-cultivation of imbibed seeds, shoot induction without (w/o) or with (w/) phosphinothricin (PPT), shoot elongation, root induction, and selection of transgenic seedlings in soil. **(E, F)** PCR-based detection of the *Bar* selection marker and InDel mutations in the *GmP34*, *GmP34h1*, and *GmP34h2* genes in T_0_ transgenic lines from GmP34 GE_CT1 **(E)** and GE_CT2 **(F)**.

Although various strategies have been developed to reduce GmP34 protein accumulation in soybean seeds—including transgene-induced gene silencing, extensive screening of natural accessions, and CRISPR/Cas9-mediated genome editing (GE) (Herman et al., 2003; Bilyeu et al., 2009; Sugano et al., 2020)—these methods have exclusively targeted the *GmP34* gene. To concurrently eliminate GmP34 along with its closely related homologs, GmP34h1 and GmP34h2, we employed the CRISPR/Cas9 system and designed three guide RNAs (gRNAs) using CRISPR-P v2.0 and RGEN tools, referred to as gRNA1, gRNA2, and gRNA3. Among them, gRNA1 and gRNA3 were designed to target all three *GmP34* homologs, whereas gRNA2 specifically targeted *GmP34* and *GmP34h1* (**Figures 3A, B**). Using these gRNAs, we constructed two multiplex genome-editing vectors in the pECO201 backbone: one expressing gRNA1 and gRNA2 to target *GmP34* and *GmP34h1*, and another expressing gRNA1 and gRNA3 to simultaneously target all three homologs. These constructs were designated GmP34 GE Common Target 1 (GmP34 GE_CT1) and Common Target 2 (GmP34 GE_CT2), respectively (**Figure 3C**).

The embryonic axis of soybean W82 was inoculated with *Agrobacterium* strains carrying either the GmP34 GE_CT1 or GmP34 GE_CT2 construct, and transgenic T_0_ plants were selected using phosphinothricin (PPT) (**Figure 3D**). Fifteen T_0_ plants were obtained for each construct, and stable integration of the T-DNA into the genome was confirmed via *Bar* gene PCR (**Figures 3E, F**). To characterize mutation patterns in each T_0_ line, the targeted genomic regions of the *GmP34* homologs were analyzed using InDel PCR and targeted deep sequencing. InDel PCR analysis revealed that T_0_ lines harboring the GmP34 GE_CT1 construct exhibited a range of deletions in the *GmP34* gene. Notably, lines #8 and #10 showed simultaneous deletions in both *GmP34* and *GmP34h1* (**Figure 3E**). Deep sequencing confirmed these findings, revealing multiple mutation types—including deletions and substitutions—with one or two predominant mutation patterns in both *GmP34* and *GmP34h1* in lines #8 and #10 (**Supplementary Figure 2A**). In the case of GmP34 GE_CT2 T_0_ lines, InDel PCR identified clear deletions in *GmP34* and *GmP34h1* in lines #1 and #14, respectively, while no distinct deletion patterns were observed in *GmP34h2* (**Figure 3F**). Sequencing analysis of CT2 line #1 showed a predominant 61-nucleotide deletion in *GmP34*, while *GmP34h1* and *GmP34h2* contained only small deletions (1–4 nucleotides) that were indistinguishable from the W82 control by InDel PCR. Similarly, CT2 line #14 exhibited a 76-nucleotide deletion in *GmP34h1*, along with minor deletions in *GmP34* and *GmP34h2* as the primary mutation patterns (**Supplementary Figure 2B**). Based on these InDel PCR and sequencing results, we selected CT1 lines #8 and #10, which carried mutations in *GmP34* and *GmP34h1*, and CT2 lines #1 and #14, which harbored mutations in all three *GmP34* homologs. These T_0_ lines were subsequently advanced to the next generation.

### 3.4 Identification of T-DNA-free *GmP34* single-mutant lines

**Figure 4:**
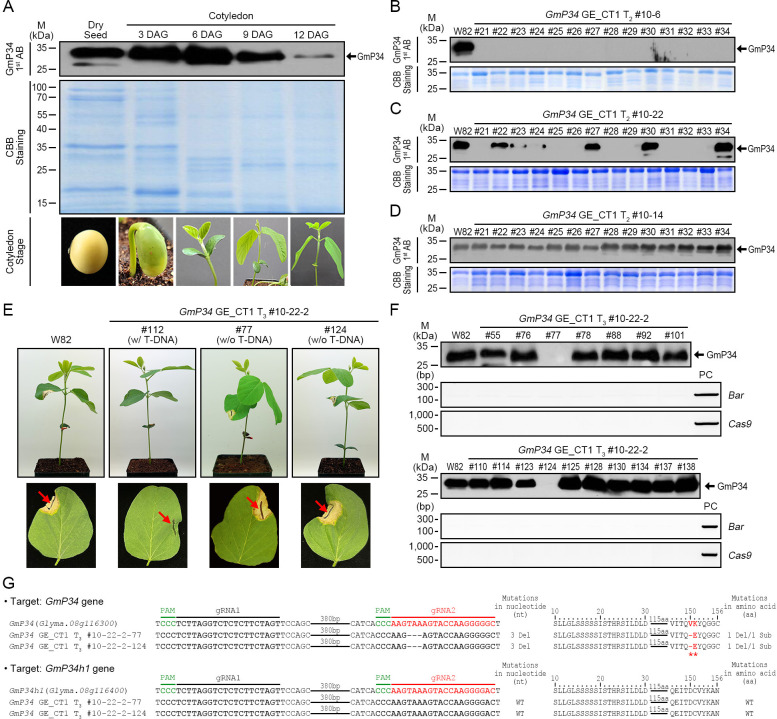
Molecular characterization of *GmP34* genome-edited (GE)_CT1 lines. **(A)** Western blot analysis of GmP34 protein levels in W82 dry seeds and cotyledons at various days after germination (DAG). **(B-D)** Detection of GmP34 protein in T_2_ progeny of *GmP34* GE_CT1 lines by western blotting. **(E)** Phosphinothricin (PPT) leaf painting assay on T_3_ seedlings from *GmP34* GE_CT1 lines. Red arrows indicate the sites of PPT application on unifoliolate leaves. **(F)** Combined western blot and PCR analyses of T3 lines, using a polyclonal anti-GmP34 antibody and gene-specific primers for *BAR* and *Cas9*. PC, positive control. **(G)** Targeted deep sequencing analysis of *GmP34* and *GmP34h1* loci in T-DNA-free T_3_ lines (#10-22-2–77 and #10-22-2-124). PAM sequences are highlighted in green. Target sequences for gRNA1 and gRNA2 are shown in bold black and red, respectively. Deletions at both the nucleotide and amino acid levels are indicated by dashes. Red asterisks mark amino acid deletions or substitutions.

Using the GmP34 GE_CT1 T1 plants (#8 and #10), we performed *Bar* PCR, InDel PCR, and targeted deep sequencing. Based on this screening, line #10—characterized by a single T-DNA insertion and higher editing efficiency (data not shown)—was selected and advanced to the T_2_ generation. We then screened T_2_ progeny (#10–1 to #10-28) for GmP34 protein expression via western blotting. Three days after sowing, when *GmP34* protein is still detectable, a small portion of the cotyledons was sampled for protein analysis (**Figure 4A**). Western blot analysis revealed that none of the #10–6 progeny expressed detectable levels of GmP34 protein in their cotyledons (**Figure 4B**). In contrast, the #10–22 progeny showed variable GmP34 protein expression (**Figure 4C**), whereas all #10–14 progeny exhibited clear GmP34 protein signals (**Figure 4D**). Subsequent *Bar* PCR analysis of these lines indicated that #10–6 was T-DNA homozygous, #10–22 was heterozygous, and #10–14 lacked the T-DNA insertion (null) (data not shown).

To generate T-DNA-free mutants for the *GmP34* homologs, we employed two approaches: (1) advancing T-DNA heterozygous lines to identify segregating T-DNA-free progeny and (2) backcrossing T-DNA homozygous lines with W82 plants and screening the F_2_ (BC_1_F_2_) generation. For the first strategy, we selected the T-DNA heterozygous *GmP34* GE_CT1 T_2_ line #10-22–2 based on *Bar* PCR and TaqMan PCR analyses and obtained T_3_ seeds (data not shown). From 3-day-old germinating T_3_ seedlings, a small portion of the cotyledons was sampled to assess GmP34 protein levels by western blotting. To identify T-DNA-free lines, we applied PPT solution to the leaves of further-grown T_3_ seedlings and selected individuals exhibiting a PPT-sensitive necrosis phenotype (**Figure 4E**). Among these, we screened for *GmP34*-null individuals using western blotting and ultimately identified two T-DNA-free GmP34-null lines: #10-22-2–77 and #10-22-2-124 (**Figure 4F**). Targeted deep sequencing revealed that both lines carried an identical 3-nucleotide deletion in *GmP34*, resulting in a single amino acid deletion (Val) and a substitution (Lys to Glu) at positions 150 and 151, respectively. No mutations were detected in the *GmP34h1* gene (**Figure 4G**). These results demonstrate the successful isolation of T-DNA-free *GmP34* single mutant lines.

### 3.5 Identification of T-DNA-free *GmP34* and *GmP34h1* double-mutant lines

**Figure 5:**
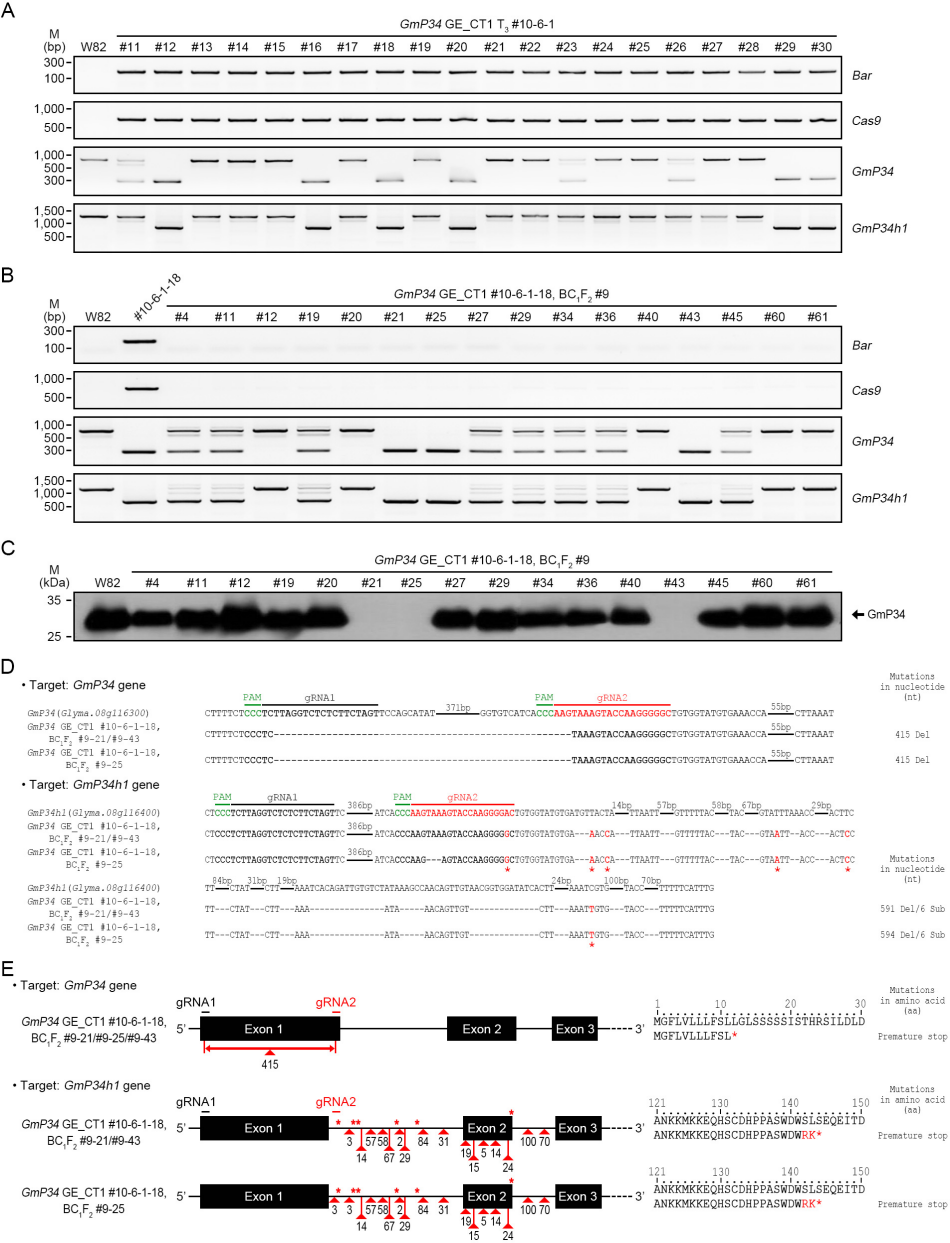
Characterization of *GmP34* GE_CT1 BC_1_F_2_ mutant lines. **(A, B)** Genomic PCR detection of *Bar* and *Cas9*, along with InDel PCR analysis of *GmP34* and *GmP34h1* genes in *GmP34* GE_CT1 T_3_ #10-6-1 **(A)** and BC_1_F_2_ #9 lines **(B)**. The backcross parent, *GmP34* GE_CT1 T_3_ #10-6-1-18, was included as a control f. **(C)** Western blot detection of GmP34 protein in T-DNA-free BC_1_F_2_ #9 lines. Total proteins were extracted from 3-day-old cotyledons of W82 and BC_1_F_2_ #9 lines, and GmP34 expression was analyzed using an anti-GmP34 antibody. **(D)** Targeted deep sequencing of *GmP34* and *GmP34h1* in T-DNA-free BC_1_F_2_ lines (#9-21, #9-25, and #9-43). PAM sequences are highlighted in green. Target sequences for gRNA1 and gRNA2 are shown in bold black and red, respectively. Nucleotide deletions are indicated by dashes; red asterisks indicate substitutions. **(E)** Summary of DNA mutations and their predicted protein-level consequences. Red triangles indicate deletion sites in *GmP34*, *GmP34h1*, and *GmP34h2* genes in lines #9-21, #9-25, and #9-43. Red asterisks mark nucleotide substitutions and premature stop codons; substituted amino acids are shown in red.

As the second strategy, we analyzed T_3_ lines derived from the T-DNA homozygous *GmP34* GE_CT1 #10-6–1 line. All T_3_ plants were confirmed to carry T-DNA based on *Bar* and *Cas9* PCR. InDel PCR analysis revealed that six lines (#12, 16, 18, 20, 29, and 30) exhibited deletions in both *GmP34* and *GmP34h1* genes (**Figure 5A**). Among these, line #10-6-1–18 was selected for backcrossing with W82, and a BC_1_F_2_ population was generated. T-DNA-free individuals were initially identified through PPT leaf painting and subsequently confirmed by *Bar* and *Cas9* PCR (**Figure 5B**). InDel PCR further revealed that three BC_1_F_2_ lines (#9-21, #9-25, and #9-43) harbored homozygous deletion patterns in both *GmP34* and *GmP34h1* identical to those of the parental #10-6-1–18 line (**Figure 5B**), confirming that they were T-DNA-free *GmP34/GmP34h1* double mutants. Western blot analysis supported these findings, showing the complete absence of GmP34 protein in cotyledons of all three lines (**Figure 5C**). Targeted deep sequencing revealed that each line carried a 415-nucleotide deletion in the first exon of *GmP34*, resulting in a frameshift and a premature stop codon at the 12th amino acid position (**Figures 5D, E**). Editing in the *GmP34h1* gene was more complex: lines #9–21 and #9–43 harbored a 591-nucleotide deletion spanning the 3’ region of the gRNA2 target site, encompassing the first intron, second exon, and second intron, along with six nucleotide substitutions (**Figures 5D, E**). Line #9–25 exhibited the same pattern with an additional 3-nucleotide deletion in the first intron (**Figure 5D**). Despite these differences, both editing types led to frameshift mutations and a premature stop codon at amino acid position 144 of the GmP34h1 protein (**Figure 5E**).

**Figure 6:**
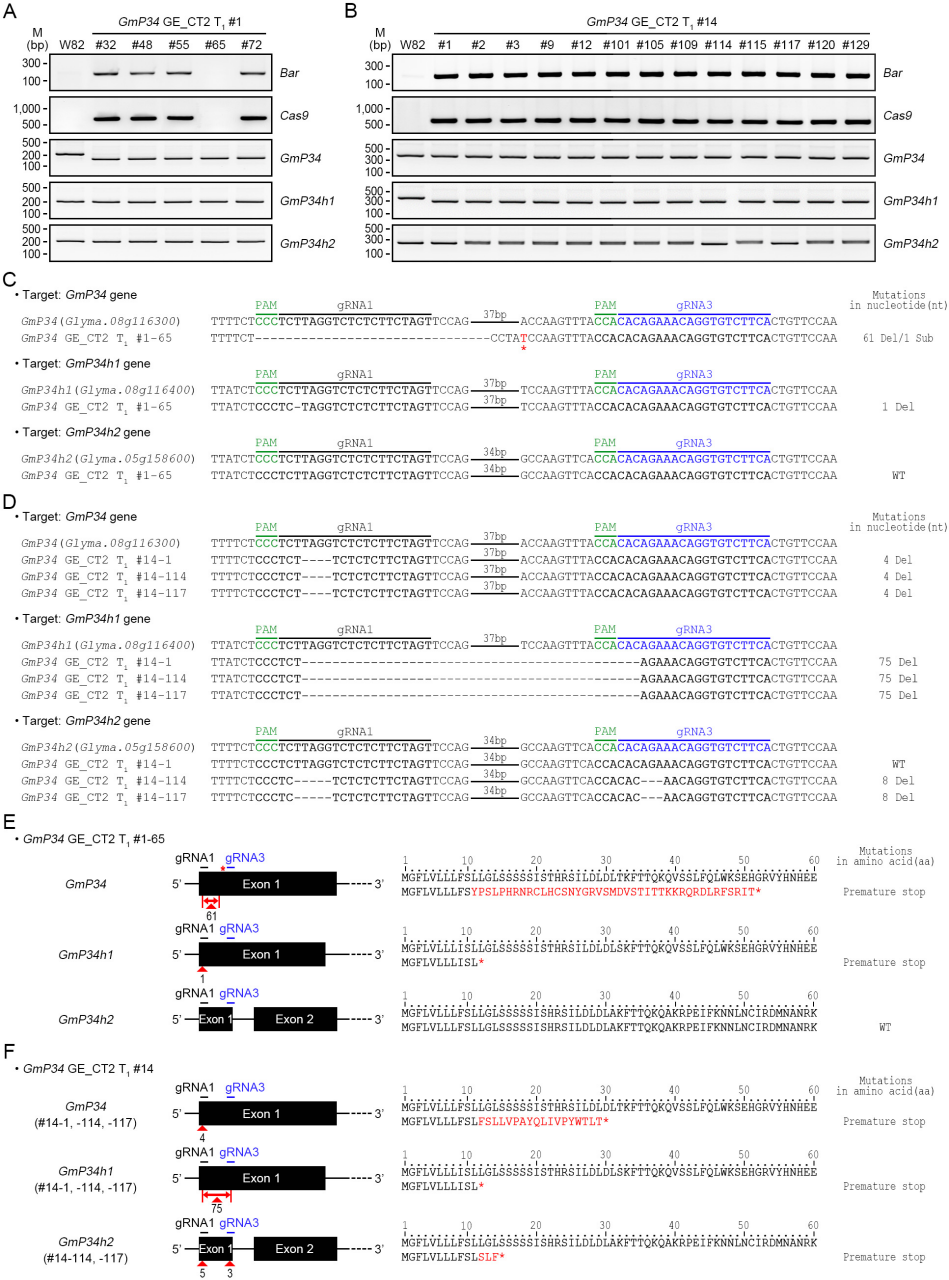
Molecular characterization of *GmP34* GE_CT2 lines. **(A, B)** Genomic PCR detection of *Bar* and *Cas9*, and InDel PCR analysis of *GmP34* homologs in GE_CT2 T_1_ lines #1 **(A)** and #14 **(B)**. **(C, D)** Targeted deep sequencing of *GmP34, GmP34h1*, and *GmP34h2* in T-DNA-free lines CT2_T_1_ #1-65 **(C)** and CT2_T_1_ #14-1, #14-114, and #14-117 **(D)**. PAM sequences are highlighted in green. Target sequences for gRNA1 and gRNA3 are shown in bold black and blue, respectively. Nucleotide deletions are indicated by dashes; red asterisks indicate substitutions. **(E, F)** Summary of nucleotide mutations and corresponding protein-level changes in #1-65 **(E)** and CT2_T_1_ #14 lines **(F)**. Red triangles indicate deletion sites in *GmP34*, *GmP34h1*, and *GmP34h2* genes. Red asterisks denote nucleotide substitutions and resulting premature stop codons. Substituted amino acids are shown in red.

Additional T-DNA-free *GmP34* and *GmP34h1* double mutants were identified through the analysis of *GmP34* GE_CT2 T_1_ lines. Western blot analysis of T_1_ seedlings from CT2 #1 and #14 lines revealed 19 and 28 GmP34 protein-null individuals, respectively (**Supplementary Figure 3**). Among these, one T-DNA-free line, designated #1-65, was identified in the CT2 #1 progeny using *Bar* and *Cas9* PCR (**Figure 6A**). InDel PCR and targeted deep sequencing showed that this line harbored a 61-nucleotide deletion along with a single-nucleotide substitution in the *GmP34* gene and a one-nucleotide deletion in the *GmP34h1* gene (**Figures 6A, C**). These mutations introduced premature stop codons at the 52nd and 12th amino acid positions of the GmP34 and GmP34h1 proteins, respectively (**Figure 6E**). No sequence alterations were observed in the *GmP34h2* gene (**Figures 6C, E**).

In contrast, *Bar* and *Cas9* PCR analyses of GmP34 protein-null CT2 #14 T_1_ seedlings indicated the absence of T-DNA-free individuals in this population (**Figure 6B**). Subsequent InDel PCR and targeted deep sequencing analyses revealed that the CT2 T_1_ #14–1 line harbored 4- and 75-nucleotide deletions in the *GmP34* and *GmP34h1* genes, respectively. These mutations introduced premature stop codons at the 30th and 12th amino acid positions of the GmP34 and GmP34h1 proteins, respectively (**Figures 6D, F**). To obtain T-DNA-free mutants, this line was backcrossed with W82 plants. Among 192 BC_1_F_2_ progeny derived from the CT2 T_1_ #14–1 line, seven T-DNA-free individuals were identified by PPT leaf painting and further confirmed by *Bar* and *Cas9* PCR (**Supplementary Figure 4A**). Western blot analysis of these lines revealed two GmP34 protein-null lines, #7–109 and #7-138 (**Supplementary Figure 4B**). InDel PCR analysis confirmed that both lines carried homozygous deletions in the *GmP34* and *GmP34h1* genes (**Supplementary Figure 4C**), and targeted deep sequencing validated the presence of the same 4- and 75-nucleotide deletions identified in the original #14–1 line (**Supplementary Figure 4D**), resulting in premature stop codons at the same amino acid positions (**Supplementary Figure 4E**). No mutations were detected in the *GmP34h2* gene. Collectively, these findings led to the identification of two additional T-DNA-free *GmP34/GmP34h1* double mutants: CT2 T_1_ #14–1 BC_1_F_2_ #7–109 and #7-138. In summary, we successfully generated six independent T-DNA-free *GmP34*/*GmP34h1* double-mutant lines: three from the BC_1_F_2_ population of the *GmP34* GE_CT1 #10-6-1–18 line (#9-21, #9-43, and #9-25), one from the *GmP34* GE_CT2 T_1_ line (#1-65), and two from the BC_1_F_2_ population of the *GmP34* GE_CT2 #14–1 line (#7–109 and #7-138).

### 3.6 Identification of *GmP34*, *GmP34h1*, and *GmP34h2* triple-mutant lines

Targeted deep sequencing revealed that two CT2 T_1_ lines, #14–114 and #14–117 carried mutations in all three *GmP34* homologs—*GmP34*, *GmP34h1*, and *GmP34h2*. Notably, the two lines exhibited identical mutation patterns, consisting of 4-, 75-, and 8-nucleotide deletions in the *GmP34*, *GmP34h1*, and *GmP34h2* genes, respectively (**Figure 6D**). These deletions caused frameshift mutations, which introduced premature stop codons at the 30th, 12th, and 15th amino acid positions of the GmP34, GmP34h1, and GmP34h2 proteins, respectively (**Figure 6F**). To generate T-DNA-free triple mutants, both lines are currently being backcrossed with W82 plants.

Taking all results together, we summarized the GE status of the three *GmP34* homologous genes in **Table 1**. Through CRISPR/Cas9-mediated GE, we successfully generated *GmP34* single mutants, *GmP34*/*GmP34h1* double mutants, and *GmP34*/*GmP34h1*/*GmP34h2* triple mutants. From the screening of the *GmP34* GE_CT1 T_3_ population, we identified two T-DNA-free *GmP34* single-mutant lines, #10-22-2–77 and #10-22-2-124. A total of six T-DNA-free *GmP34*/*GmP34h1* double-mutant lines were obtained—one directly from the *GmP34* GE_CT2 T_1_ population (#1-65), and five from BC_1_F_2_ populations derived from CT1 #10-6-1-18 (#9-21, #9-25, and #9-43) and CT2 #14-1 (#7–109 and #7-138) lines. In addition, we identified two *GmP34*/*GmP34h1*/*GmP34h2* triple-mutant lines (#14–114 and #14-117) from the *GmP34* GE_CT2 T_1_ population, which are currently being backcrossed with W82 to eliminate the T-DNA.

**Table 1:** Summary of the genome editing status of *GmP34* homologs.

Genome Editing Lines	T-DNA	*GmP34* (*Glyma.08g116300*)		*GmP34h1* (*Glyma.08g116400*)		*GmP34h2* (*Glyma.05g158600*)	
		Mutations in nucleotide (nt)	Mutations in amino acid (aa)	Mutations in nucleotide (nt)	Mutations in amino acid (aa)	Mutations in nucleotide (nt)	Mutations in amino acid (aa)
CT1 T_3_ #10-22-2-77, CT1 T_3_ #10-22-2-124	Free	3 nt Del^1)^	1 aa Del & 1 aa Sub^2)^	–	–	–	–
CT1 T_3_ #10-6-1-18, BC_1_F_2_ #9-21, #9-43	Free	415 nt Del	Premature stop	591 nt Del & 6 nt Sub	Premature stop	–	–
CT1 T_3_ #10-6-1-18, BC_1_F_2_ #9-25	Free	415 nt Del	Premature stop	594 nt Del & 6 nt Sub	Premature stop	–	–
CT2 T_1_ #1-65	Free	61 nt Del & 1 nt Sub	Premature stop	1 nt Del	Premature stop	–	–
CT2 T_1_ #14-1, BC_1_F_2_ #7-109, #7-138	Free	4 nt Del	Premature stop	75 nt Del	Premature stop	–	–
CT2 T_1_ #14-114^*^	○	4 nt Del	Premature stop	75 nt Del	Premature stop	8 nt Del	Premature stop
CT2 T_1_ #14-117^*^	○	4 nt Del	Premature stop	75 nt Del	Premature stop	8 nt Del	Premature stop

^1),2)^Del and Sub indicate deletion and substitution mutations, respectively.

^*^Backcrossing with W82 is in progress.

## 4 Discussion

Soybean is an important dietary protein source, though its allergenic properties continue to pose challenges for both consumers and food producers. Among the allergenic seed proteins, GmP34, a papain-like cysteine protease, has been recognized as a key allergen despite its relatively low presence in seeds (Ogawa et al., 1993; Helm et al., 2000). Various strategies, including co-suppression (Herman et al., 2003), screening diverse soybean accessions (Joseph et al., 2006), and CRISPR/Cas9-based genome editing (Sugano et al., 2020; Adachi et al., 2021), have been employed to eliminate GmP34 from seeds. However, most of these efforts have focused solely on the *GmP34* gene, potentially overlooking allergenic effects from its homologous counterparts. In this study, we extensively characterized two closely related GmP34 homologs, GmP34h1, and GmP34h2, which share strong sequence similarity with GmP34, including conserved IgE-binding regions and functional motifs (**Figure 1**). The concurrent expression of all three genes during seed maturation suggests functional overlap and their collective role in contributing to soybean allergenicity (**Figure 2**). To simultaneously suppress all three *GmP34* homologs, we engineered two multiplex CRISPR/Cas9 constructs capable of targeting *GmP34*, *GmP34h1*, and *GmP34h2*. This approach generated a range of genome-edited lines, including T-DNA-free single, T-DNA-free double, and triple mutants. Notably, two triple mutants exhibited frameshift mutations introducing premature stop codons in all three genes, underscoring the efficiency of the multiplex method (**Figure 6**). This represents the first report of concurrent editing of multiple allergen-related gene families in soybean, addressing gene redundancy due to duplication—a hallmark of the soybean genome (Schmutz et al., 2010)—and illustrating the power of multiplex genome editing in complex polyploid crops.

Reverse genetics techniques such as T-DNA and transposon-mediated insertional mutagenesis, along with RNA interference (RNAi)-based gene silencing, have long been instrumental in uncovering gene functions and enhancing crop traits (Alonso and Ecker, 2006). However, these methods come with notable drawbacks. Insertional mutagenesis often leads to random or biased genomic insertions, reducing its effectiveness for comprehensive functional analyses (Krysan et al., 2002). RNAi, while widely used, is susceptible to off-target effects and may not achieve complete gene suppression (Neumeier and Meister, 2021). Critically, neither approach is well-suited for targeting multiple genes simultaneously. This limitation is especially problematic when attempting to edit members of multigene families or tandemly repeated genes—an issue compounded in polyploid crops like soybean, where gene redundancy is common (Alonso and Ecker, 2006). In contrast, recent advancements in genome editing tools—such as zinc finger nucleases, transcription activator-like effector nucleases, and the CRISPR/Cas9 system—have enabled precise, efficient, and multiplex gene modifications (Gao, 2021). Of these, CRISPR/Cas9 has rapidly emerged as the preferred platform for genetic improvement in many crops, including soybean (Bao et al., 2019; Baek et al., 2022; Nerkar et al., 2022). In this study, we designed multiplex CRISPR/Cas9 constructs to simultaneously target three homologous allergen genes in soybean: *GmP34* (Glyma.08G116300), *GmP34h1* (Glyma.08G116400), and *GmP34h2* (Glyma.05G158600). Notably, *GmP34* and *GmP34h1* are tandem duplicates on chromosome 8, while GmP34h2 resides on chromosome 5. Using this system, we successfully generated soybean lines with concurrent mutations in the tandemly duplicated *GmP34* and *GmP34h1* genes (**Figures 5**, **6**; **Table 1**). Additionally, we produced triple mutant lines carrying edits in all three genes—*GmP34*, *GmP34h1*, and *GmP34h2* (**Figure 6**; **Table 1**)—as well as *GmP34* single mutants (**Figure 4**; **Table 1**). These findings underscore the precision and versatility of CRISPR/Cas9 genome editing, highlighting its potential to overcome the inherent constraints of traditional mutagenesis and breeding, and positioning it as a powerful approach for improving complex polyploid crops such as soybean.

Our genome editing approach produced a wide array of edited soybean lines, including single, double, and triple mutants. All mutant lines exhibited frameshift mutations that led to premature stop codons in the *GmP34* homolog genes, except for the *GmP34* single mutants (**Table 1**). Two of these single mutant lines (CT1 T_3_ #10-22-2–77 and #10-22-2-124) carried the same 3-nucleotide deletion, which caused the removal of a valine residue and a lysine-to-glutamate substitution at positions 150 and 151 of the GmP34 protein, respectively (**Figure 4G**). Sequencing confirmed that no additional mutations were present in the *GmP34* gene in these lines (data not shown). Interestingly, western blotting with a polyclonal anti-GmP34 antibody failed to detect the GmP34 protein in these mutants (**Figure 4F**), indicating that these two amino acid alterations may substantially compromise GmP34 protein stability or disrupt its epitope. These observations suggest that the deleted and substituted residues are likely critical for maintaining the structural integrity or functional role of GmP34. Further studies exploring their impact on protein stability and allergenic potential are warranted. In an effort to isolate *GmP34* single mutants with frameshift mutations, we extensively screened BC_1_F_2_ progeny derived from CT1 T_3_ #10-6-1–18 and CT2 T_1_ #14–1 lines using InDel PCR, targeted deep sequencing, and western blotting. However, such mutants were not recovered (data not shown), likely due to suppressed recombination between the closely linked *GmP34* and *GmP34h1* genes during backcrossing. We are currently performing further backcrosses of triple mutant lines CT2 T_1_ #14–114 and #14–117 with the W82 cultivar, aiming to isolate T-DNA-free single and triple mutants with frameshift mutations that may segregate in subsequent generations through recombination.

A gene editing system represents a distinct form of genetic modification that entails the deliberate alteration of an organism’s genome (Ahmad et al., 2023). Traditionally, genetically modified (GM) plants—those incorporating exogenous transgenes via biotechnological methods—have been classified as genetically modified organisms (GMOs) by scientists and regulatory bodies (Ahmad et al., 2023). Since 1986, the United States Department of Agriculture (USDA) has regulated GMOs under the Coordinated Framework. In 2020, the USDA implemented significant updates to its biotechnology regulations (Code of Federal Regulations, 7 CFR 340), reflecting advancements in genome editing technologies (Tachikawa and Matsuo, 2024). Notably, certain genome-edited organisms may be exempt from regulatory oversight if they meet one of five specified criteria—one of which states that the modification must involve cellular repair of a targeted DNA break without the use of an exogenous transgene (Tachikawa and Matsuo, 2024). To address regulatory concerns related to GMOs and to enable the integration of hypoallergenic soybean lines into elite germplasm and consumer-sensitive products such as baby food and infant formula, it is crucial to eliminate T-DNA from genome-edited lines. To generate T-DNA-free mutants, we employed two distinct strategies and successfully obtained T-DNA-free lines for both *GmP34* single mutants and *GmP34*/*GmP34h1* double mutants (**Table 1**). The *GmP34* single mutant lines (CT1 T_3_ #10-22-2–77 and #10-22-2-124) and the *GmP34*/*GmP34h1* double mutant line (CT2 T_1_ #1-65) were isolated by selecting T-DNA-free segregants from progeny of heterozygous T-DNA-containing plants. In contrast, the remaining T-DNA-free double mutants were identified among BC_1_F_2_ progeny resulting from backcrosses of CT1 T_3_ #10-6-1–18 and CT2 T_1_ #14–1 with wild-type W82. Additionally, we are currently backcrossing the triple mutant lines CT2 T_1_ #14–114 and #14–117 with W82 in an effort to obtain T-DNA-free triple mutants. We consider the backcrossing approach followed by BC_1_F_2_ screening to be more advantageous than direct selection from segregating heterozygous lines, particularly for minimizing potential off-target effects of genome editing. Although CRISPR/Cas9 is renowned for its specificity, it can still generate unintended edits at off-target loci (Graham et al., 2020). Backcrossing with wild-type plants can aid in eliminating such mutations through segregation. Future research should aim to quantify the frequency of off-target mutations in CRISPR-edited soybean lines and to assess the effectiveness of backcrossing in removing them—using whole-genome sequencing as a comprehensive evaluation tool.

In this study, we present a foundational advance toward the development of hypoallergenic soybean cultivars. Looking ahead, it will be essential to assess the allergenic potential of the single, double, and triple-mutant lines relative to wild-type soybean using immunoreactivity assays with sera from soy-allergic individuals. In summary, our study presents a robust and scalable genome editing strategy for reducing seed allergenicity in soybean by simultaneously targeting multiple *GmP34* homologs. This strategy not only provides a promising pathway for allergen reduction in soybean but also serves as a valuable model for reducing allergenicity in other polyploid crops. Moreover, it highlights the potential of multiplex CRISPR/Cas9-mediated editing to effectively address gene redundancy, a common challenge in modern crop improvement.

## Data Availability

The original contributions presented in the study are included in the article/**Supplementary Material**. Further inquiries can be directed to the corresponding authors.
