# Supplementary material for: CRISPR/Cas9-mediated simultaneous targeting of GmP34 and its homologs produces T-DNA-free soybean mutants with reduced allergenic potential
Source: Front Plant Sci. 2025 Aug 1;16:1612747. doi: 10.3389/fpls.2025.1612747 (PMC12354366; doi:10.3389/fpls.2025.1612747)
Supplement: Supplementary file 1 [file Supplementaryfile1.docx]

Supplementary Material

# Supplementary Tables

**Supplementary Table 1.** **Features and putative off-targets of guide RNAs used in this study.**

|  | **gRNA sequence** | **GC content (%)** | **Off-target site** | **MM^1^** | **Off-score^2^** | **Gene** | **Position of off-target^3^** |
| --- | --- | --- | --- | --- | --- | --- | --- |
| **gRNA1** | ACTAGAAGAGAGACCTAAGA | 40 | ACTAGAAGAGAGACCTAAGAGGG | 0 | 1.000 | Glyma.08g116300  (GmP34) | exon |
|  |  |  | ACTAGAAGAGAGACCTAAGAGGG | 0 | 1.000 | Glyma.08g116400  (GmP34h1) | exon |
|  |  |  | ACTAGAAGAGAGACCTAAGAGGG | 0 | 1.000 | Glyma.05g158600  (GmP34h2) | intron |
|  |  |  | ACCAGAAGAAAAACCTAAGAAGG | 3 | 0.373 |  | Intergenic |
|  |  |  | ACCAGAAGAAAAACCTAAGAAGG | 3 | 0.373 |  | Intergenic |
|  |  |  | ACTCAAAGAGAAACCTAAGATGG | 3 | 0.285 |  | Intergenic |
| **gRNA2** | GCCCCCTTGGTACTTTACTT | 50 | GCCCCCTTGGTACTTTACTTGGG | 0 | 1.000 | Glyma.08g116300  (GmP34) | exon |
|  |  |  | GTCCCCTTGGTACTTTACTTGGG | 1 | 0.909 | Glyma.08g116400  (GmP34h1) | exon |
| **gRNA3** | TGAAGACACCTGTTTCTGTG | 45 | TGAAGACACCTGTTTCTGTGTGG | 0 | 1.000 | Glyma.08g116300  (GmP34) | exon |
|  |  |  | TGAAGACACCTGTTTCTGTGTGG | 0 | 1.000 | Glyma.08g116400  (GmP34h1) | exon |
|  |  |  | TGAAGACACCTGTTTCTGTGTGG | 0 | 1.000 | Glyma.05g158600  (GmP34h2) | exon |

^1^ MM means the sequence mismatches throughout the whole genome.

^2^ Score of gRNA off-target potential.

^3^ The position of off-target is validated based on the Wm82.a2.v1 reference genome in the Phytozome V13 database.

**Supplementary Table 2. List of primers used in this study.**

| **Gene** | **Primer Name** | **Sequence (5’→3’)** | **Description** | |
| --- | --- | --- | --- | --- |
| *GmP34*  (*Glyma.08g116300*)/  *GmP34h1*  (*Glyma.08g116400*) | MG-3497 | 5’-TGCAGACTAGAAGAGAGACCTAAGA-3’ | gRNA1 | CT1 editing vector cloning |
|  | MG-3498 | 5’-AAACTCTTAGGTCTCTCTTCTAGTC-3’ |  |  |
|  | MG-3499 | 5’-TGCAGGCCCCCTTGGTACTTTACTT-3’ | gRNA2 |  |
|  | MG-3500 | 5’-AAACAAGTAAAGTACCAAGGGGGCC-3’ |  |  |
| *GmP34*  (*Glyma.08g116300*)/  *GmP34h1*  (*Glyma.08g116400*)/  *GmP34h2*  (*Glyma.05g158600*) | MG-3497 | 5’-TGCAGACTAGAAGAGAGACCTAAGA-3’ | gRNA1 | CT2 editing vector cloning |
|  | MG-3498 | 5’-AAACTCTTAGGTCTCTCTTCTAGTC-3’ |  |  |
|  | MG-3928 | 5’-GCAGTGAAGACACCTGTTTCTGTG-3’ | gRNA3 |  |
|  | MG-3929 | 5’-AAACCACAGAAACAGGTGTCTTCAC-3’ |  |  |
| *GmP34*  (*Glyma.08g116300*) | MG-3808 | 5’-CGACATGCTACAAGTGAAGTGACC-3’ | CT1  1^st^ indel PCR | Figure 5A & 5B |
|  | MG-3809 | 5’-GGTAATCTCTTTCACTAATA  AGGTTAGTTTGG-3’ |  |  |
|  | MG-3811 | 5’-ACACTCTTTCCCTACACGACGCTCT  TCCGATCTCAAGTGAAGTGACCATATC-3’ | CT1  2^nd^ indel PCR | Figure 3E |
|  | MG-3812 | 5’-GTGACTGGAGTTCAGACGTGTGCTCTTC  CGATCTATATAAAACAATTTAAGACAACTG-3’ |  |  |
| *GmP34h1*  (*Glyma.08g116400*) | MG-3810 | 5’-TGTAACCACTATATATATGAGGTGGTGC-3’ | CT1  1^st^ indel PCR | Figure 5A & 5B |
|  | MG-4413 | 5’-CCGCCAGTGTAGAAATGAAAATC-3’ |  |  |
|  | MG-3813 | 5’-ACACTCTTTCCCTACACGACGCTCTTCC  GATCTACCACTATATATATGAGGTGG-3’ | CT1  2^nd^ indel PCR | Figure 3E |
|  | MG-3769 | 5’-GTGACTGGAGTTCAGACGTGTGCTCTTC  CGATCTAACACATTTTAAATGATACC-3’ |  |  |
| *GmP34*  (*Glyma.08g116300*) | MG-3808 | 5’-CGACATGCTACAAGTGAAGTGACC-3’ | CT2  1^st^ indel PCR | |
|  | MG-3809 | 5’-GGTAATCTCTTTCACTAATAAGGT  TAGTTTGG-3’ |  |  |
|  | MG-3811 | 5’-ACACTCTTTCCCTACACGACGCTCTTC  CGATCTCAAGTGAAGTGACCATATC-3’ | CT2  2^nd^ indel PCR | Figure 3F & Figure 6B |
|  | MG-4054 | 5’-GTGACTGGAGTTCAGACGTGTGCTCTT  CCGATCTCTGTTTGCATTCATGTCC-3’ |  |  |
|  | MG-4475 | 5’-ACACTCTTTCCCTACACGACGCTCTTC  CGATCTGTTTCCTTGTGTTGCTTC-3’ |  | Figure 6A |
|  | MG-3765 | 5’-GTGACTGGAGTTCAGACGTGTGCTCTT  CCGATCTTCACTCTTCCATAGTTGG-3’ |  |  |
| *GmP34h1*  (*Glyma.08g116400*) | MG-3810 | 5’-TGTAACCACTATATATATGAGGTGGTGC-3’ | CT2  1^st^ indel PCR | |
|  | MG-3496 | 5’-CCGCCAGTGTAGAAATGAAAATC-3’ |  |  |
|  | MG-3813 | 5’-ACACTCTTTCCCTACACGACGCTCTTCC  GATCTACCACTATATATATGAGGTGG-3’ | CT2  2^nd^ indel PCR | Figure 3F & Figure 6B |
|  | MG-4055 | 5’-GTGACTGGAGTTCAGACGTGTGCTCTT  CCGATCTTGTCCCTGATATAGTTCAAG-3’ |  |  |
|  | MG-4475 | 5’-ACACTCTTTCCCTACACGACGCTCTTC  CGATCTGTTTCCTTGTGTTGCTTC-3’ |  | Figure 6A |
|  | MG-3765 | 5’-GTGACTGGAGTTCAGACGTGTGCTCTT  CCGATCTTCACTCTTCCATAGTTGG-3’ |  |  |
| *GmP34h2*  (*Glyma.05g158600*) | MG-3964 | 5’-CTCTTTTCTCTCACTAAGCATTTGGGC-3’ | CT2  1^st^ indel PCR | |
|  | MG-4056 | 5’-TGTTGATGTGTCGTGGCACATCC-3’ |  |  |
|  | MG-3811 | 5’-ACACTCTTTCCCTACACGACGCTCTTC  CGATCTCAAGTGAAGTGACCATATC-3’ | CT2  2^nd^ indel PCR | Figure 3F & Figure 6B |
|  | MG-4057 | 5’-GTGACTGGAGTTCAGACGTGTGCTCTT  CCGATCTATTCATGTCCCTGATACAG-3’ |  |  |
|  | MG-4475 | 5’-ACACTCTTTCCCTACACGACGCTCTTC  CGATCTGTTTCCTTGTGTTGCTTC-3’ |  | Figure 6A |
|  | MG-4476 | 5’-GTGACTGGAGTTCAGACGTGTGCTCTT  CCGATCTTTACTCTTCCATGGTTGG-3’ |  |  |
| *Bar* | MG-4591 | 5’-CCTTCTGAAGTCCCTGGAGG-3’ | Genomic PCR | |
|  | MG-4592 | 5’-GAAGTCCAGCTGCCAGAAAC-3’ |  |  |
| *Cas9* | MG-4326 | 5’-GACCTACAACCAGCTTTTCGA-3’ |  |  |
|  | MG-4327 | 5’-GCTTGATGAACTTGTAGAACTC-3’ |  |  |

# Supplementary Figures


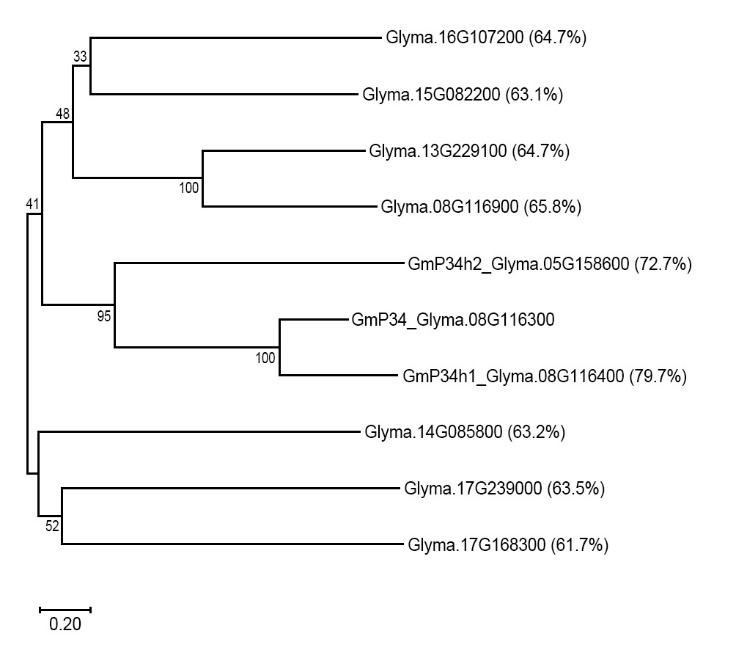


**Supplementary Figure 1. Phylogenetic analysis of soybean GmP34 homologs.**

The ten closest homologs of GmP34 were identified using Phytozome v13 (<https://phytozome-next.jgi.doe.gov/>). Evolutionary phylogenetic tree analysis was inferred using the Neighbor-Joining method in the MEGA-X program.


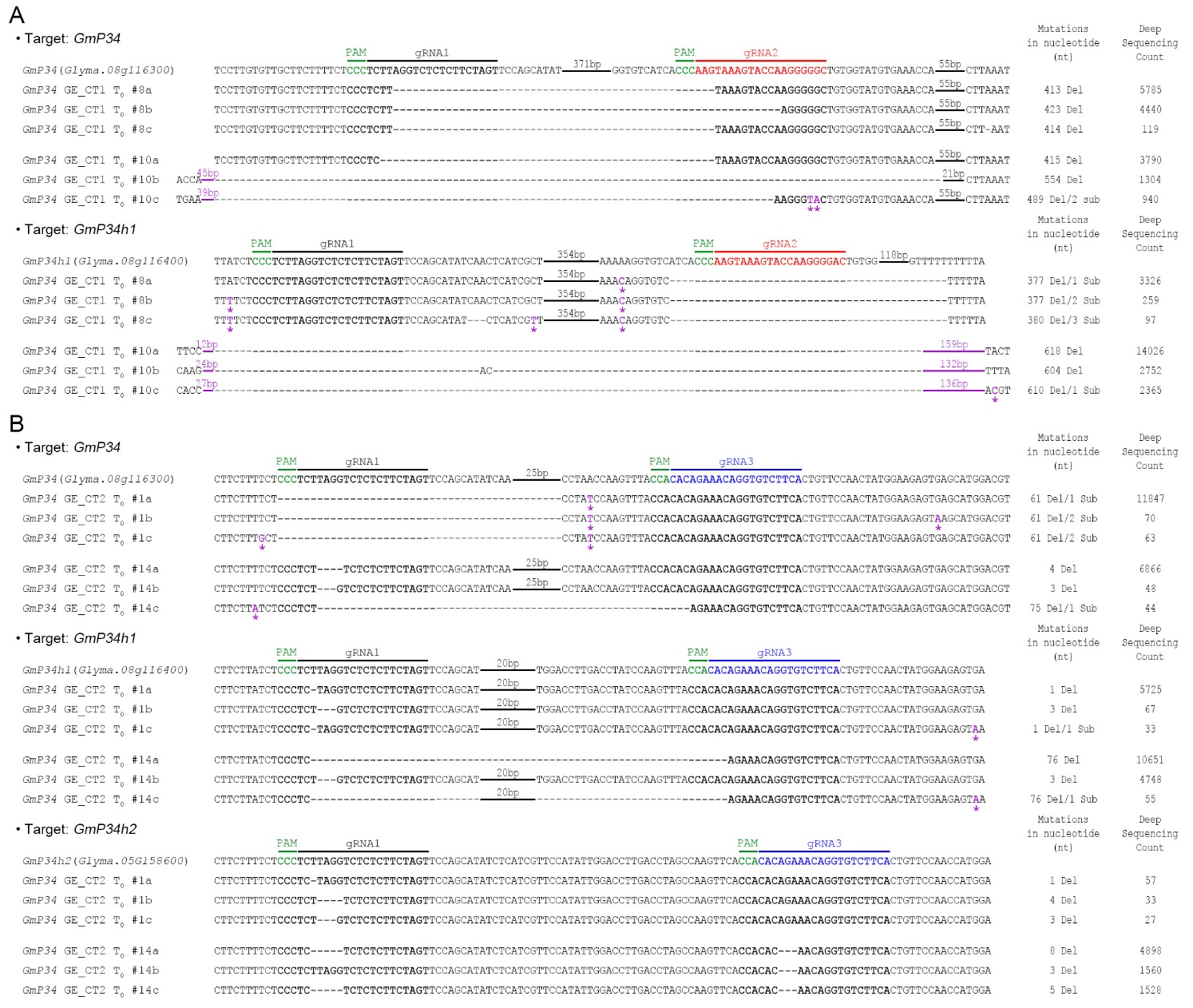


**Supplementary Figure 2. Targeted deep sequencing analysis of T_0_ genome-edited soybean lines.**

**(A, B)** Deep sequencing of *GmP34, GmP34h1*, and *GmP34h2* loci in T_0_ plants from the *GmP34* GE_CT1 **(A)** and GE_CT2 **(B)** transformation events. PAM sequences are highlighted in green. Target regions for gRNA1, gRNA2, and gRNA3 are shown in bold black, red, and blue, respectively. Nucleotide deletions are indicated by dashes and substitutions by purple asterisks or lines. Among the various genome editing outcomes, the top three mutation patterns are shown along with their read counts.


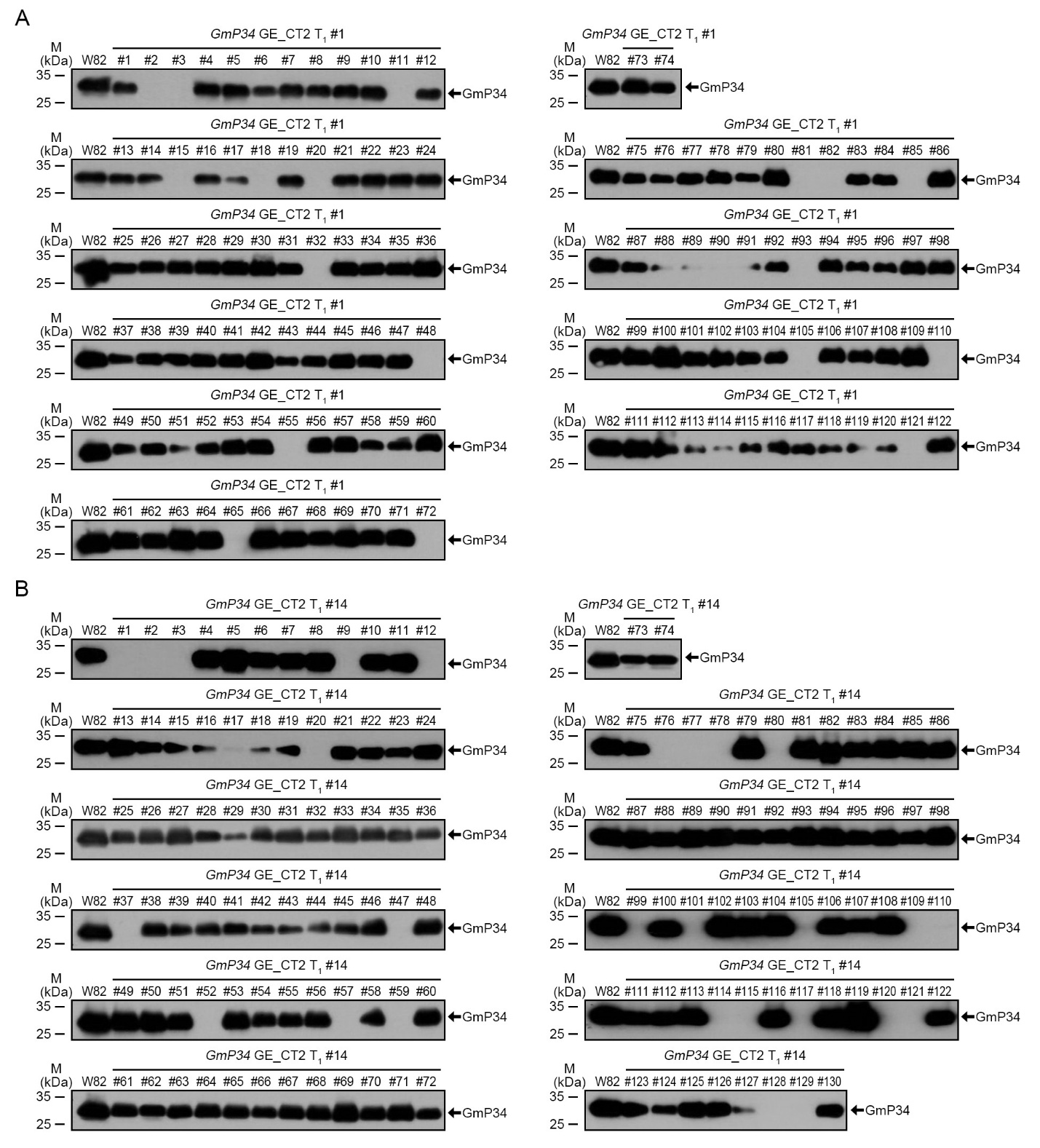


**Supplementary Figure 3. Western blot analysis of GmP34 protein levels in cotyledons of *GmP34* GE_CT2 T_1_ lines.**

**(A, B)** Total protein was extracted from 3-day-old cotyledons of wild-type W82 and *GmP34* GE_CT2 T_1_ plants (#1 and #14). One microgram of total protein per sample was analyzed by western blot using a polyclonal anti-GmP34 antibody.


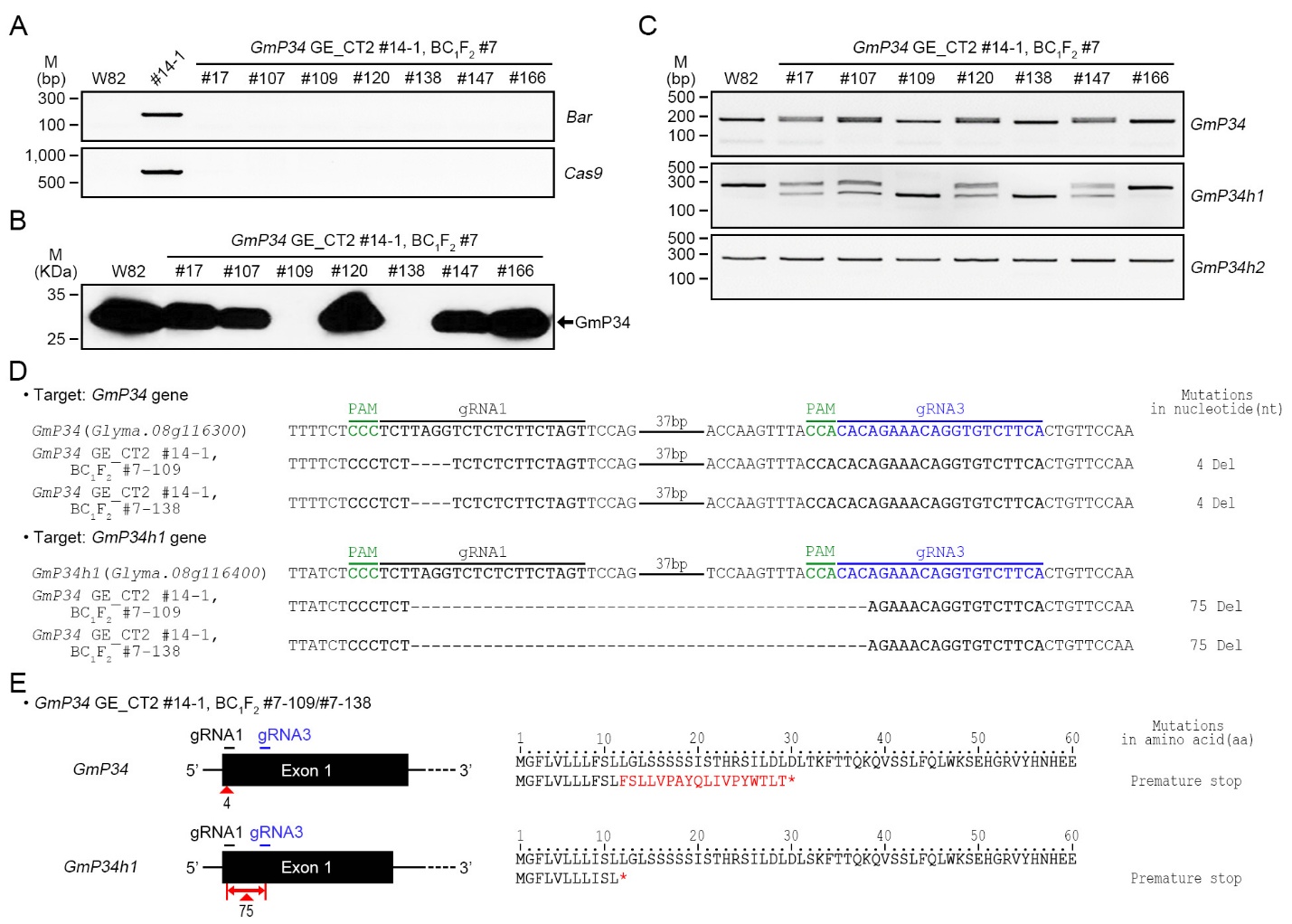


**Supplementary Figure 4. Molecular characterization of *GmP34* GE_CT2 BC_1_F_2_ #7 lines.**

**(A**) Genomic PCR analysis for the presence of the *Bar* and *Cas9* transgenes in T1 line #14-1 and derived BC_1_F_2_ #7 lines. The parental line (#14-1) served as a control. **(B)** Western blot analysis of GmP34 protein expression in T-DNA-free BC_1_F_2_ #7 lines. Total protein was extracted from 3-day-old cotyledons of W82 and BC_1_F_2_ lines. **(C)** Indel PCR analysis targeting *GmP34*, *GmP34h1*, and *GmP34h2* in BC1F2 #7 lines using gene-specific primers. **(D)** Targeted deep sequencing of *GmP34* and *GmP34h1* loci in T-DNA-free BC_1_F_2_ lines (#7-109 and #7-138). PAM sequences are highlighted in green. Target sites for gRNA1 and gRNA3 are shown in bold black and blue, respectively. Nucleotide deletions are represented by dashes. **(E)** Summary of editing outcomes, showing DNA mutations and their predicted effects on protein sequence. Red triangles indicate deletion sites; red asterisks denote premature stop codons; amino acid substitutions are marked in red.


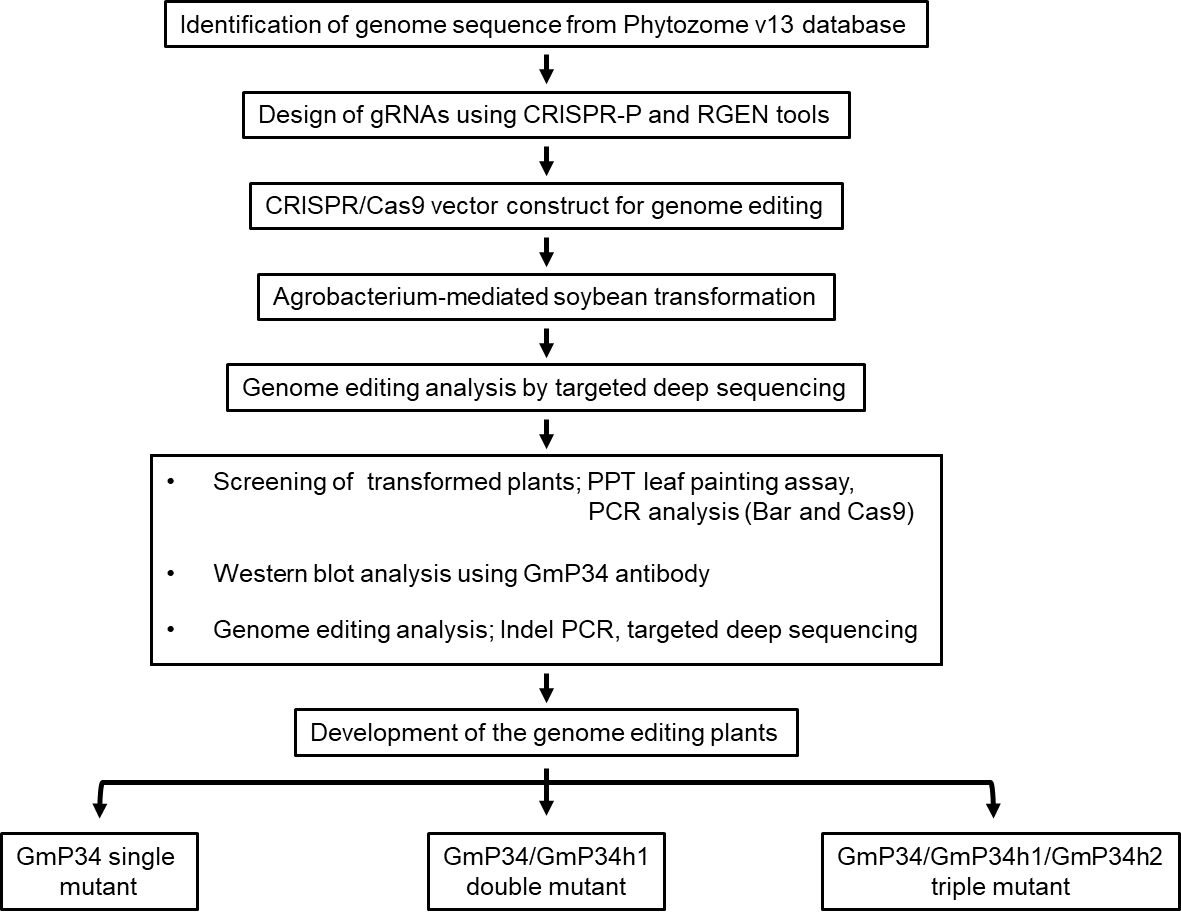


**Supplementary Figure 5. The schematic flowchart illustrates the generation of *GmP34* GE_CT1 and *GmP34* GE_CT2 genome editing plants using the CRISPR/Cas9 system.**
